# Hokkaido birth cohort study on environment and children’s health: cohort profile 2021

**DOI:** 10.1186/s12199-021-00980-y

**Published:** 2021-05-22

**Authors:** Reiko Kishi, Atsuko Ikeda-Araki, Chihiro Miyashita, Sachiko Itoh, Sumitaka Kobayashi, Yu Ait Bamai, Keiko Yamazaki, Naomi Tamura, Machiko Minatoya, Rahel Mesfin Ketema, Kritika Poudel, Ryu Miura, Hideyuki Masuda, Mariko Itoh, Takeshi Yamaguchi, Hisanori Fukunaga, Kumiko Ito, Houman Goudarzi, the members of The Hokkaido Study on Environment and Children’s Health, S. Sasaki, T. Ikeno, E. Okada, S. Nishihara, T. Kita, I. Kashino, T. Baba, T. S. Braimoh, S. Minakami, K. Cho, N. Shinohara, K. Moriya, T. Mitsui, Y. Nishimura, T. Saito, S. Suyama, T. Nomura, S. Konno, H. Matsuura, M. Ishizuka, T. Endo, F. Sata, K. Sengoku, Y. Saijo, E. Yoshioka, T. Miyamoto, M. Yuasa, J. Kajiwara, T. Hori, Y. Chisaki, T. Matsumura, F. Mizutani, J. Yamamoto, Y. Onoda, N. Sato, T. Kawai, T. Tsuboi, T. Nakajima, K. Miyake, T. Kubota

**Affiliations:** 1 Center for Environmental and Health Sciences, Hokkaido University, Kita 12, Nishi 7, Kita-ku, Sapporo, Hokkaido 060-0812 Japan; 2 WHO Collaborating Centre for Environmental Health and Prevention of Chemical Hazards, Sapporo, Japan; 3 Faculty of Health Sciences, Hokkaido University, Sapporo, Japan; 4 Graduate School of Health Sciences, Hokkaido University, Sapporo, Japan; 5 Faculty of Medicine and Graduate School of Medicine, Center for Medical Education and International Relations, Hokkaido University, Sapporo, Japan

**Keywords:** Early life, Environmental chemicals, Secular trend, Birth size, Hormones, Development, Allergies and infectious diseases, Genetic polymorphisms, Epigenetics, DOHaD

## Abstract

**Background:**

The Hokkaido Study on Environment and Children’s Health is an ongoing study consisting of two birth cohorts of different population sizes: the Sapporo cohort and the Hokkaido cohort. Our primary objectives are to (1) examine the effects that low-level environmental chemical exposures have on birth outcomes, including birth defects and growth retardation; (2) follow the development of allergies, infectious diseases, and neurobehavioral developmental disorders, as well as perform a longitudinal observation of child development; (3) identify high-risk groups based on genetic susceptibility to environmental chemicals; and (4) identify the additive effects of various chemicals, including tobacco.

**Methods:**

The purpose of this report is to provide an update on the progress of the Hokkaido Study, summarize recent results, and suggest future directions. In particular, this report provides the latest details from questionnaire surveys, face-to-face examinations, and a collection of biological specimens from children and measurements of their chemical exposures.

**Results:**

The latest findings indicate different risk factors of parental characteristics on birth outcomes and the mediating effect between socioeconomic status and children that are small for the gestational age. Maternal serum folate was not associated with birth defects. Prenatal chemical exposure and smoking were associated with birth size and growth, as well as cord blood biomarkers, such as adiponectin, leptin, thyroid, and reproductive hormones. We also found significant associations between the chemical levels and neuro development, asthma, and allergies.

**Conclusions:**

Chemical exposure to children can occur both before and after birth. Longer follow-up for children is crucial in birth cohort studies to reinforce the Developmental Origins of Health and Disease hypothesis. In contrast, considering shifts in the exposure levels due to regulation is also essential, which may also change the association to health outcomes. This study found that individual susceptibility to adverse health effects depends on the genotype. Epigenome modification of DNA methylation was also discovered, indicating the necessity of examining molecular biology perspectives. International collaborations can add a new dimension to the current knowledge and provide novel discoveries in the future.

## Background

Environmental factors play an important role in determining the health and well-being of children [1]. Windows of susceptibility during the prenatal to postnatal period govern the differentiation of each organ system and developmental stage [1]. In addition, evidence from research has indicated the importance that the fetus and early life periods have on later life, i.e., the Developmental Origins of Health and Disease (DOHaD) theory [2, 3]. The DOHaD approach initially focused on nutritional deficiency in utero and the onset of adult diseases, such as coronary heart disease. However, the DOHaD concept has been expanded to multidisciplinary fields that examine how environmental factors affect the developmental plasticity window and interact with genotype variations [4]. Thus, evidence for the integration of the “Environment” into the DOHaD has become important, especially for the prevention of diseases and dysfunctions in terms of clinical and public health implications [4]. Accordingly, previous studies have increasingly recognized the importance of birth cohort studies for the examination of the early life environment and follow-up of children, which has resulted in the establishment of several birth cohort studies from the 1980s to 2000s [5, 6].

In Japan, the Hokkaido Study on Environment and Children’s Health: Malformation, Development, and Allergy (the Hokkaido Study) is an ongoing birth cohort study since 2001 [7–9]. The Hokkaido Study consists of two cohorts: (1) The Sapporo cohort recruited pregnant women during 2002-2005 and has performed detailed child neurobehavioral examinations. (2) The Hokkaido cohort recruited pregnant women during their first trimester from the entirety of Hokkaido Prefecture, Japan, during 2003-2012. As the Hokkaido cohort included more than 20,000 women, we could detect relatively rare and/or low prevalence outcomes, such as birth defects, and developmental disorders, such as autism spectrum disorder (ASD). We have previously published three cohort profiles. In the first profile paper, we included detailed birth information for the Sapporo cohort, whereas recruitment for the Hokkaido cohort was still ongoing at that stage [7]. The second paper was published 10 years after the launch of the cohort study [8]. The second paper investigated the follow-up profiles for children up to 7 and 8 years old in the Sapporo and Hokkaido cohorts, respectively. The second paper also introduced findings related to the concentrations of toxic equivalents (TEQ) of dioxins and other congeners of polychlorinated dibenzofurans (PCDF) and polychlorinated dibenzodioxins (PCDD). Maternal genetic polymorphisms significantly modify the dioxin accumulation in the human body, in addition to the association between specific single nucleotide polymorphism (SNP) holders for mothers who smoked tobacco during their pregnancies [8]. In the latest profile paper [9], we reported the basic characteristics of the finalized cohort populations, together with a comparison of those remaining in the cohorts and those who had not been followed up at birth. Detailed environmental chemical exposure levels suggested that the chemical exposure levels were relatively low. However, there were associations with health outcomes, such as a reduced birth size, decrease or increase in neonatal hormone levels, delays in neurobehavioral development and behavior up to 5 years of age, increase or decrease in asthma and allergies, and an increase in infectious disease occurrences up to 4 years of age. The third paper also introduced the newly added concept of case-cohort study design and epigenetics.

Four years have passed since the most recent profile paper. This coincides with the follow-up stage with the shift to pubertal age. Children are not only exposed to environmental chemicals in utero, but also continue to be exposed after birth. Questionnaire survey and face-to-face examination becomes important as for limitation survey. Thus, this report aims to provide an update on the progress of the Hokkaido Study. In particular, this paper focuses on the (1) profiles of the children with further follow-ups until pubertal age, (2) exposure assessments of the children, and (3) summaries of our recent findings. This paper also discusses the importance of longer follow-up periods and future research directions.

## Methods

### Study area and participant enrollment

Our previous cohort profile papers describe the details of the Hokkaido Study [7–9]. The Hokkaido study on the Environment and Children’s Health is an ongoing cohort study that began in 2002. The study consists of two prospective birth cohorts: the Sapporo (Toho hospital) cohort and the Hokkaido (large-scale) cohort.

Enrollment in the Sapporo cohort was conducted from July 2002 to October 2005. The subjects included 514 pregnant women who enrolled at 23–35 weeks of gestation and delivered at Toho hospital. All subjects were residents of Sapporo City or the surrounding areas. From February 2003 to March 2012, the Hokkaido (large-scale) cohort conducted enrollment during early pregnancy (gestational age of < 13 weeks) for women that visited one of the associated hospitals or clinics in the study area for prenatal health care in the maternity unit. This cohort consists of 20,926 pregnant women. In total, 37 hospitals and clinics in Hokkaido prefecture participated in the study (the names of the hospitals are listed at the end of this paper).

The study was conducted with the informed and written consent of all participants. The Institutional Ethical Board for Human Gene and Genome studies at Hokkaido University Center for Environmental and Health Sciences (CEHS) and Hokkaido University Graduate School of Medicine approved the study protocol. Table 1 summarizes the details of the two cohorts (i.e., the Sapporo cohort and Hokkaido cohort).

**Table 1:** Summary of the birth cohort study

	Hokkaido cohort	Sapporo cohort
Recruitment	2003-2012	2002-2005
Participates	20,926 (37 clinics/hospitals)	514 (1 hospital)
Objectives	1. To find the effects of perinatal environmental factors, especially chemical exposure at low levels, on child health, including congenital anomalies, growth retardation, birth size, allergies, neurodevelopment, growth, and puberty. 2. To evaluate the prevalence of allergic diseases, developmental and neurobehavioral disorders. 3. To identify a high-risk group classified by genetic susceptibility (SNPs) and investigate trans-generational epigenetic effects of environmental chemicals. 4. To provide scientific evidence for health policies based on human epidemiological data.	

### Follow-up survey

#### Follow-up survey until birth

At the first trimester, participants completed the baseline survey on maternal and paternal characteristics. Maternal peripheral blood samples were taken during the first-mid and third trimester, and postpartum hospitalization. Cord blood was collected at birth. Medical birth records from the delivery hospital were collected for birth weight, height, sex, and other medical conditions.

#### After birth follow-up survey

Data on the body size, allergic symptoms, and history of infectious diseases for the child participants were collected at 1, 2, 4, and 7 years of age in the follow-up surveys (Table 2). Childhood wheezing and eczema symptoms were evaluated using a modified section of the Japanese version of the International Study of Asthma and Allergies in Childhood (ISAAC) Phase Three questionnaire. Urine samples from the children and house dust were collected at 7 years of age. Data regarding child neurodevelopment were collected at 5, 6, and 8 years of age. Data regarding social economic status, including the duration of parental educational and household income, were collected during pregnancy, and at 6 and 7 years of age. The Strengths and Difficulties Questionnaire (SDQ) and Social Communication Questionnaire (SCQ) were used to assess behavioral problems at 5 years of age. The attention-deficit hyperactivity disorder rating scale (ADHD-RS) was used to assess suspected ADHD symptoms at 6 and 8 years of age. Data regarding the height and weight during each school year were transcribed by the parents using the health records provided by the elementary and secondary schools. Data regarding the Tanner staging and onset of puberty in the children, as well as urine and tap water samples, were collected at 12 years of age.

**Table 2:** Follow-up studies (Sapporo and Hokkaido cohorts)

	Questionnaire Survey			Face-to-face examinations	Specimen/sample collections	Exposure measurements
	Neurobehavioral development	Allergy/infections	Anthropometric measurements/puberty			
**Sapporo cohort**						
6–7 months	EES			BSID-II, FTII,		
1.5 years	EES	ISAAC, ATS-DLD, infections	Physical growth	BSID-II, DDST,		
3.5 years	CBCL, EES	ISAAC, infections	Physical growth	K-ABC, WAIS-R,		
7 years	CBCL, J-PSAI, 2D/4D	ISAAC, infections	Physical growth	WISC-III, WCST-KFS,		
12 years	Tanner staging, onset		Tanner staging, onset of puberty	Event-related brain potentials: 11–14 years	Urine of children (9–12)	Neonicotinoids
13 years			Physical growth during elementary school			
**Hokkaido cohort**						
4 months	Physical Growth					
1 year		ISAAC, ATS-DLD, infections	Physical growth			
1.5 years	KIDS, M-CHAT					
2 years		ISAAC, infections	Physical growth			
3 years	KIDS, SDQ					
4 years		ISAAC, infections	Physical growth			
5 years	SDQ, DCDQ					
6 years	ADHD-RS, ASQ (SCQ)					
7 years		ISAAC	Health checkup data	Home visit	Urine of children/house dust	Urine: Phthalates, PFRs, Bisphenols, Alternative plasticizers, Neonicotinoids, and organophosphate pesticides House dust: Phthalates, alternative plasticizers, and PFRs
8 years	ADHD-RS, Conners 3P,		CBCL, WISC-IV			
9–11 years		ISAAC, history of vaccination		Medical examination phase 1 (Blood pressure, anthropometric examination, FeNO, ISAAC)	Peripheral blood/urine of children	Urine: Phthalates, PFRs, Bisphenols, Alternative plasticizers Blood: PFAS
12 years	SDQ / KIDSCREEN: 8–17 years		Tanner staging, onset of puberty	Event-Related Brain Potentials: 11–14 years	Urine of children/tap water	
13 years			Physical growth during elementary school			
16 years		ISAAC	Physical growth during junior high school	Medical examination phase 2 (Tanner staging, Blood pressure, anthropometric examination, Gripping power, 2D/4D, Consumer-product-use, Personal care products): 14–17 years	Peripheral blood/urine of children/house dust	

*2D/4D* 2nd and 4th digits ratios, *ADHD-RS* Attention Deficit Hyperactivity Disorder-Rating Scale, *ASQ* Autism Screening Questionnaire, *ATS-DLD* American Thoracic Society-Division of Lung Disease, *M-CHAT* Modified Checklist for Autism in Toddlers, *BSID-II* Bayley Scales of Infant Development second edition, *CBCL* Child Behavior Checklist, *Conners 3P* Conner’s 3rd Edition for Parents, *DCDQ* Developmental Coordination Disorder Questionnaire, *DDST* Denver Developmental Screening Tests, *EES* Evaluation of Environmental Stimulation, *FeNO* Fractional exhaled nitric oxide, *FTII* Fagan Test of Infant Intelligence, *ISAAC* International Study of Asthma and Allergies in Childhood, *J-PSAI* Japanese Pre-School Activities Inventory, *K-ABC* Kaufman Assessment Battery for Children, *KIDS* Kinder Infant Development Scale, *PFRs* phosphate flame retardants, *SCQ* Social Communication Questionnaire, *SDQ* Strengths and Difficulties Questionnaire, *WAIS-R* Wechsler Adult Intelligence Scale-Revised, *WISC-III* Wechsler Intelligence Scale for Children third edition, *WCST-KFS* Wisconsin Card Sorting Test (Keio Version), *WISC-IV* Wechsler Intelligence Scale for Children fourth edition

### Face-to-face health check and home visit

In the Sapporo cohort, face-to-face examinations to assess child neurodevelopment, such as BSID-II, K-ABC, and WISC-III, were conducted at 1.5 and 3.5, 3.5, and 7 years of age, respectively.

In the Hokkaido cohort, for 7-year-old children living in the Sapporo area, a home visit survey was conducted to investigate the associations between indoor environments and the health of the participating children. Well-trained investigators visited the homes and collected vacuumed house dust in the living rooms and child bedrooms using a handheld vacuum cleaner. House dust samples were collected from two parts of each room: the floor surface, surfaces 35 cm above the floor, and the surface of objects more than 35 cm from the floor. The morning void urine of the children was also collected. To collect long-term dust samples, a dust-collection box (all surfaces were wrapped in aluminum foil) was stored for 6 months on the top of an object with an approximate height of 140–180 cm. Six months later, the mothers wrapped the stored dust samples in aluminum foil and sent them to the CEHS.

We conducted face-to-face assessments, e.g., clinical examinations related to asthma and allergies, to collect detailed data that could not be assessed from the questionnaires. We recruited 1930 children, aged 9–12, living in Sapporo city and its suburbs between September 2017 and March 2020. Among them, 428 children agreed to visit pediatricians for an anthropometric examination (height and weight) and medical examination by a doctor, as well as a measurement of the fractional exhaled nitric oxide (FeNO) and collection of blood and urine samples. Immediately before the examination, we directly informed participants of the purpose and methods of the study, in addition to notifying them of the results. Examinations were carried out in 12 cooperating pediatric clinics. The Tanner staging, blood pressure, anthropometric examination, gripping power, and 2nd and 4th digits ratios (2D/4D) were examined during the phase 2 clinical examination. The use of consumer and personal care products were evaluated at 14–17 years of age. Samples of peripheral blood, urine, and house dust were also collected.

### Exposure measurements

The maternal serum folate at the first-mid trimester and plasma cotinine at the third trimester were measured. The prenatal environmental chemical exposure evaluation detected persistent organic pollutants (POPs), including PCDDs, PCDFs, polychlorinated biphenyls (PCBs) [10–12], hydroxylated polychlorinated biphenyl (OH-PCB) [13], 11 perfluoroalkyl substances (PFAS) [14, 15], and organochlorine pesticides (OCPs) [16] in the maternal plasma samples. For the evaluation of chemicals with short half-lives, di(2-ethylhexyl) phthalate (DEHP) in maternal blood and bisphenol A in cord blood were detected [17].

To assess current child exposure to environmental chemicals, urine samples of the children were collected at ages 7 and 12. These urine samples were used in the internal exposure assessment of compounds characterized by biologically short half-lives, such as phthalate, alternative plasticizers, bisphenols, phosphate flame retardants (PFRs), and insecticides [18–21]. Morning void urine samples were collected via uniform urinary collection kits made of polypropylene. Each urine sample was shipped to CEHS via a cool shipment service, dispensed into glass tubes or vials in the lab, and stored in − 30°C until analysis. Biological markers, such as cotinine, 8-hydroxy-2’-deoxyguanosine (8-OHdG) as oxidative stress marker, hexanoyl-lysine (HEL) and 4-hydroxynonenal (HNE) as inflammation markers, and creatinine, were also measured in the urine samples at age 7 [20]. For the external exposure assessment, phthalates, alternative plasticizers, and organophosphorus flame retardants were measured from the house dust samples [22–24], which were collected by the mothers of 7-year-old children using normal household vacuum cleaners with uniform dust bags and shipped to CEHS. After removing the paper, food, hair, and unwanted objects from the dust samples, they were sieved with a 150-μm mesh, dispensed into a glass tube, and stored in freezers at −30 °C until analysis.

### Genetics and epigenetics

Genotyping of single nucleotide polymorphisms (SNPs) was performed using allelic discrimination assays, with fluorogenic probes and 5′ nuclease (TaqMan) (Applied Biosystems, Foster, CA, USA), or a nanofluidic integrated fluidic circuit-based genotyping system for the medium-throughput multiplexing of 96 individual human DNA samples and 96 individual SNP assays (Dynamic Array, Fluidigm Corporation, South San Francisco, CA, USA) [25–27]. Patterns of the offspring DNA methylation in the cord blood were analyzed by pyrosequencing using a PyroMark Q24 system (Qiagen, Hilden, Germany) [28] or quantified using an Infinium HumanMethylation450 BeadChip (Illumina, San Diego, CA, USA) according to the manufacturer’s protocol [29, 30].

## Results

### Outcomes and related factors: updated results

#### Birth size and socioeconomic status

**Fig. 1:**
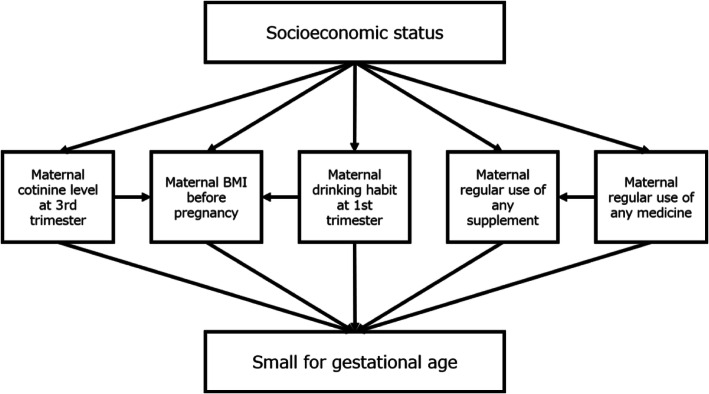
Directed acyclic graph for the mediating factors between parental SES and small for gestational age

We identified the different parental risk factors for preterm-birth (PTB), very-low-birth weight (VLBW), and small for gestational age at full-term (term-SGA) groups. An older parental age (≥ 35) and assisted reproductive technology (ART) were risk factors for PTB (< 37 weeks) and VLBW (< 1500 g). Maternal alcohol consumption during pregnancy and a lower parental educational level (< 16 years) were risk factors for term-SGA. A lower maternal pre-pregnancy body mass index (BMI) (< 18.5 kg/m^2^) was a risk factor for PTB and term-SGA [31]. In addition, we described the mediating factors between parental socioeconomic status (SES) and SGA on a directed acyclic graph (Fig. 1). Based on the results of structure equation modeling, we observed the independent mediating effect of maternal pre-pregnancy BMI, smoking, and alcohol consumption during pregnancy on low SES and, consequently, SGA, with the additional mediating pathway of SES on smoking and low BMI on SGA [32]. We found that the risk of hypertensive disorders during pregnancy (HDP) was higher among older mothers (≥35 years), with high BMI (≥25.0kg/m^2^), multiple pregnancies, and those who underwent in vitro fertilization (IVF). Furthermore, the mothers with HDP had an increased risk to deliver small for gestational age (SGA), preterm (PTB), and low birth weight (LBW) babies as compared to mothers with normotensive pregnancy [33].

#### First trimester prevalence of birth defects and maternal folate levels

All birth defects after 12 weeks of gestation were examined, including 55 marker anomalies. The prevalence of all birth defects was 18.9/1000 births in the Hokkaido cohort. The proportion of patients with birth defects delivered within 12–21 weeks of gestation was 9.4% of all patients with birth defects. Among those with a congenital malformation of the nervous system, 39% were delivered before 22 weeks of gestation [34].

Moreover, the association between the maternal serum folate levels in the first trimester (median: 16.5 nmol/L) and birth defects was examined. Based on the log-transformed folate level, the probability ratio for any birth defect, ventricular septal defect, and cleft lips was 0.99 (95% CI, 0.74–1.32), 0.63 (95% CI, 0.30–1.33), and 4.10 (95% CI, 0.96–17.58), respectively. There were no significant associations between the first-trimester maternal serum folate and the risk of birth defects [35].

#### Prenatal chemical exposure, birth size, growth, and cord adiponectin and leptin as predictors

Bisphenol A and phthalates, along with other environmental obesogenic chemicals, have particularly played a role in the obesity epidemic. Examining the disruption of metabolic regulators in newborns, possibly induced by exposure to environmental chemicals during fetal development, is challenging. One technique to detect and monitor metabolic dysfunctions is using metabolic-related biomarkers, e.g., adipokines such as adiponectin and leptin [36]. In the Hokkaido cohort, we investigated the cord adipokine levels associated with exposure to these chemicals (Table 3).

**Table 3:** Prenatal chemical exposure and association with adipocytokines at birth

	Biological specimens and exposure level (ng/ml, median, IQR)	Findings	Ref.
**PFOS**	Maternal blood at 2nd–3rd trimester 5.1 (3.7–6.7)	Positively associated with total adiponectin levels (*β*=0.12, 95% CI 0.01, 0.22)	[37]
**MEHP**	Maternal blood at 2nd–3rd trimester 8.81 (5.45–13.3)	Positively associated with adiponectin level among boys (*β* = 4.63, 95% CI 0.77, 8.94). Negatively associated with leptin level among girls (*β* = −0.31, 95% CI −0.52, −0.10).	[38]
**MEHP**	Maternal blood at 1st trimester 1.50 (0.82–9.35)	Inversely associated with the leptin levels (β=0.08, 95%CI −0.14, −0.03).	[39]
**MnBP**	Maternal blood at 1st trimester 26.0 (17.0–37.0)	Inversely associated with leptin level (*β* = −0.12, 95%CI −0.24, 0.00)	[39]
**MiBP**	Maternal blood at 1st trimester 6.95 (4.60–9.58)	Inversely associated with leptin level (*β*=-0.14, 95%CI −0.27, −0.01).	[39]
**BPA**	Maternal blood at 1st trimester 0.060 (0.023–0.250)	Negatively associated with the leptin level (*β* = −0.06, 95% confidence interval [CI] −0.11, −0.01) and positively associated with the high-molecular-weight adiponectin level (*β* = 0.03, 95%CI 0.00, 0.06).	[39]

In the Sapporo cohort, prenatal exposure to perfluorooctanesulfonic acid (PFOS) and DEHP in maternal blood at second to third trimester was positively associated with the cord adiponectin levels [37, 38]. Exposure to PFOS and perfluorooctanoic acid (PFOA) was negatively associated with PI and birth weight, respectively [37]. Exposure to DEHP was negatively associated with birth size (ponderal index: PI) [38]. Our findings suggest that prenatal exposure to phthalates and PFAS may alter cord adipokine levels and may decrease birth size.

In the Hokkaido cohort, the levels of bisphenol A, mono-isobutyl phthalate (MiBP), mono-n-butyl phthalate (MnBP), mono-(2-ethylhexyl) phthalate (MEHP), and ∑DEHPm (sum of MEHP and MECPP) measured in maternal serum at the first trimester were negatively associated with the cord leptin level [39]. These results indicate that prenatal exposure to bisphenol A and certain phthalates may modify fetal leptin levels.

#### Disruption of thyroid hormones in mothers and children

In Table 4, we summarized the associations between chemical exposures in utero and thyroid hormone levels at birth. Our results for the Sapporo cohort showed that maternal dioxins, PCBs, and hydroxylated PCBs (OH-PCBs) were positively associated with both maternal and neonatal free thyroxine (FT4) [40, 41]. OCPs was also positively associated with neonatal FT4, whereas it was negatively associated with maternal FT4 [42]. PFOS was negatively and positively associated with maternal thyroid-stimulating hormone (TSH) and neonatal TSH, respectively [43]. We also investigated the associations of maternal DEHP and Bisphenol A with neonatal thyroid hormone (TH); however, no significant associations were observed [44, 45]. Our results suggest that exposure to POPs during pregnancy affects both the maternal and neonatal TH levels, whereas compounds with a short half-life have little effect on TH disruption.

**Table 4:** Associations between environmental chemicals and the maternal and neonatal thyroid hormone levels found in the Hokkaido cohort

Specimen for exposure assessment	Specimen for outcome assessment	Exposure	Sample size	Maternal TH	Neonatal TH	Results	Ref.
**Sapporo cohort**							
Maternal blood (2nd–3rd trimester)	Heel pick (4.3 (median) days old)	Dioxin	358	FT4↑	FT4↑	PCBs and Non-ortho PCBs were positively associated with maternal FT4. Coplanar PCBs and Dioxin (TEQ) were associated with increased neonatal FT4, and the association was more significant among boys.	[40]
		PCBs	386 mothers 410 neonates	FT4↑	FT4↑		
		OH-PCBs	222	FT4↑	FT4↑	Maternal OH-PCB levels were positively associated with maternal and neonatal FT4 levels.	[41]
		OCPs	333	FT4↓	FT4↑	Maternal DDE, DDT, and dieldrin levels were inversely associated with maternal FT4, while maternal cis-nonachlor and mirex were positively associated with neonatal FT4.	[42]
		PFOS and PFOA	392	TSH↓	TSH↑	Maternal PFOS levels were inversely correlated with maternal serum TSH and positively associated with infant serum TSH levels, whereas maternal PFOA showed no significant relationship with TSH or FT4 levels among mothers and infants.	[43]
		DEHP	328		→	No association.	[44]
Cord blood		Bisphenol A	285		→	No association.	[45]
**Hokkaido cohort**							
Maternal blood (1st trimester)	Cord blood	PFAS	701	FT3↑, TPOAb↓	Boys: TSH ↑, FT3↓ [TA(+)] TSH↓, TgAb↓[TA(−)] Girls: FT4 ↓, TgAb↑ [TA(+)] TSH↓, FT3↑[TA(−)]	Maternal PFAS levels were positively associated with maternal FT4 levels and inversely associated with maternal TPOAb. Among boys, some maternal PFAS were associated with higher TSH, lower FT3 levels and TgAb maternal TA-negative group [TA(-)], while PFDA was associated with lower TSH in maternal TA-positive group [TA(+)]. Among girls, some PFAS of mothers showed associations with lower TSH and higher FT3 in maternal [TA(-)] , while PFDoDA was associated with lower FT4 and higherTgAb in maternal [TA(+)].	[46]

↑/↓ Arrows indicate the following: ↓ inversely associated (*p* < 0.05); ↑ positively associated (*p* < 0.05); and → no significant association. Blanks indicate not measured

In the Hokkaido cohort, we found that maternal PFAS was associated with thyroid antibodies (TA), as well as TH [46]. In addition, our results indicate that the maternal TA status may be an effect modifier in the relationship of PFAS with neonatal THs and thyroglobulin antibody (TgAb). These findings suggest that maternal exposure to these chemicals during pregnancy, even at background environmental levels, disrupts not only the maternal TH circulation but also neonatal TH circulation.

#### Disruption of reproductive hormone levels at birth

As shown in Table 5, we summarized the associations between chemical exposures in utero and reproductive hormone levels at birth. In our manuscript [47], we noted an increase in the maternal PCDD/PCDF and dioxin-like PCBs related to decreased testosterone/estradiol (T/E2) ratios, and steroid hormone binding globulin (SHBG) and inhibin B levels, as well as increased adrenal androgens/glucocorticoid (AA/GC) ratios and follicle stimulating hormone (FSH) and dehydroepiandrosterone (DHEA) levels, in the male cord blood samples. However, we noted an increase in the maternal mono-ortho polychlorinated biphenyls related to increased cortisol, cortisone, and SHBG levels, as well as decreased DHEA levels and AA/GC ratios, in female cord blood samples [47]. OCPs were positively correlated with DHEA, FSH, and the adrenal androgen/glucocorticoid and FSH/inhibin B ratios among males. Sex hormone-binding globulin and prolactin (PRL) showed an inverse association with the OCPs. Among females, the linear regression model showed that only p,p′-dichlorodiphenyltrichloroethane was negatively associated with the level of DHEA and the adrenal androgen/glucocorticoid ratio, but was positively associated with cortisone levels [48]. Maternal PFOS levels had a significantly positive association with estradiol (E2), but a negative association with T/E2, progesterone (P4), and inhibin B. The PFOA levels were positively associated with the inhibin B levels in males. Among females, although there were no significant associations between the PFOA levels and the reproductive hormone levels of the female infants [49], there were significant negative associations between the PFOS levels and the P4 and PRL levels. For glucocorticoids and androgenic hormones, the maternal PFOS level was negatively associated with the cortisol and cortisone levels, whereas they were positively associated with the DHEA levels. The maternal PFOA level was negatively associated with the DHEA level [50]. These results suggest that exposure to environmental chemicals in utero may cause an endocrine disrupting effect on fetal synthesis and the secretion of steroid and reproductive hormones.

**Table 5:** Associations between environmental chemicals and the steroid and reproductive hormones at birth

Exposures	P4	T	E2	T/E2	SHBG	LH	FSH	Inhibin B	INSL3	Others	ref.
Prenatal											
Dioxins (Male)	→	→	→	→	→	→	→	↓	→		[47]
OCPs (Both male and female)	→	↓	→	↑	↓	→	→	→	→	DHEA↑, T/androstenedione↓, prolactin↓	[48]
PFOS (Male)	↓	→	→	↓	→	→	→	↓	↓		[49, 50]
PFOA (Male)	→	→	→	→	→	→	→	↑	→	DHEA↓, cortisol↓, cortisone↓	
PFOS (Female)	↓	→	→	→	↓	NIL	NIL	NIL	NIL	Prolactin ↓	
PFOA (Female)	→	→	→	→	→	NIL	NIL	NIL	NIL	DHEA↑, cortisol↓, cortisone↓	
DEHP (Male)	↓	→	→	↓	→	→	→	↓	↓		[17, 51]
DEHP (Both Male and female)	↓	→	→	→	→	→	→	→	→	Cortisol↓, cortisone↓, cortisol/cortisone↓, glucocorticoid/adrenal androgen↓, DHEA/androstenedione↑	
At birth											
BPA in cord blood (Male)	↑	↑	→	→	→	→	→	→	→		[45]

↑/↓ Arrows indicate the following: ↓ inversely associated (*p* < 0.05); ↑ positively associated (*p* < 0.05); and → no significant association. NIL indicates not examined in the study due to low detection of hormones among females

The concentrations of cortisone, as well as the androstenedione/DHEA and cortisone/cortisol ratios, in the cord blood were positively correlated with the birth weight for males, but not for females [52].

#### Recent findings related to neurodevelopment

In the Sapporo birth cohort, for neurodevelopment, prenatal exposure to cis-heptachlor epoxide, an organochlorine pesticide, had a negative association with mental development at 18 months, but not at 6 months of age [53]. Prenatal exposure to total non-ortho PCBs was negatively associated with the cognitive ability in males while several dioxin-like compounds were positively associated with the achievement score (i.e., the basis of intelligence in all children at 42 months) [54]. Prenatal exposure to MECPP at the 1st trimester was associated with increase conduct problem risk [55].

In the Hokkaido birth cohort, for developmental disorder symptoms, active maternal smoking, as determined by the levels of maternal plasma cotinine, contributed to an increased risk in a child’s total difficulties and hyperactivity/inattention at a pre-school age [56]. Smoking during pregnancy was also the main factor associated with motor coordination problems in preschool-aged children [57]. As described in subsection “Prenatal chemical exposure, birth size, growth, and cord adiponectin and leptin as predictors”, the association between the decreased hyperactivity/inattention and increased leptin suggested that cord blood adipokines may be a predictor of hyperactivity/inattention problems at a preschool age [58].

#### Effects of environmental chemical exposures on allergies and infectious diseases up to 7 years of age

The prevalence of ISAAC-defined wheeze, eczema, and rhino-conjunctivitis at 2, 4, and 7 years of age were 19.3%, 17.8%, and 4.4% [59]; 18.7%, 19.0%, and 5.4 % [60]; and 11.9%, 21.0%, and 11.3 % [61], respectively. This indicates an increasing transition to the prevalence of eczema and rhino-conjunctivitis, but a decreasing transition to wheeze.

The effects that prenatal exposure to PFAS have on childhood allergies and infectious diseases have been previously investigated longitudinally at 2, 4, and 7 years of age [59–61]. Based on our findings, prenatal exposure to PFAS was consistently associated with reduced risks of childhood rhino-conjunctivitis and eczema at each investigated age [59–61]. In contrast, prenatal exposure to PFAS was associated with an increased risk of infectious diseases, such as pneumonia and respiratory syncytium virus infections, among children without siblings [61, 62]. Cord blood IgE levels decreased significantly with high maternal PFOA concentrations among female infants [63]. An increasing risk of infectious diseases and decreasing risk of allergies indicate the immunomodulatory and immunotoxicity effects of PFAS, especially PFOS (C8), PFOA (C8), and longer carbon-chain PFAS, such as PFDA (C10), PFUnDA (C11), and PFDoDA (C12). The characterization of longer half-lives, higher PFAS bioaccumulation, and the higher transplacental transfer efficiency of PFAS are considered to have an enhanced potential for adverse effects on humans.

Prenatal exposure to dioxon-like compound was positively associated with the frequency of wheezing in children aged up to 7 years. At 3.5 years, males showed negative associations between maternal dioxin-like compound concentrations and cord blood IgE, as well as the frequency of wheezing, but females did not [64].

Prenatal exposure to DEHP was negatively associated with cord blood IgE levels and increased risks of food allergies, as reported by the mothers, and infectious diseases, such as otitis media and chicken pox up to 7 years of age [65].

We also considered other factors that affect childhood allergies. Maternal serum cotinine during pregnancy was associated with an increased risk of wheezing in children at ages 1, 2, and 4, but the association disappeared at age 7 [66]. In contrast, maternal cotinine levels were negatively associated with the prevalence of eczema in children at 7 years of age. Maternal pre-pregnancy BMI, not the BMIs of the children, had a positive association with wheeze and a negative association with eczema in 7-year-old children. None of the examined maternal factors were associated with rhino-conjunctivitis [66].

### Measurements of postnatal exposure levels

To investigate postnatal internal exposure to shorter biological half-life environmental chemicals among children, 10 phthalate metabolites [21], 13 PFR metabolites [19, 20], and 7 bisphenols [18] were measured in 400 urine samples from 7-year-old children between 2012 and 2017, as listed in Table 2.

All of the targeted phthalate metabolites were detected in approximately > 95% of the samples. The most abundant metabolite was mono(2-ethyl-5-carboxypentyl) phthalate (MECPP), followed by MnBP and mono(2-ethyl-5-hydroxyhexyl) phthalate. The least abundant was MiNP, followed by MBzP and cx-MiNP [21]. Half (7/14) of the PFR metabolites were detected in > 50% of the samples. The most abundant PFR metabolite was DPHP, followed by BCIPHIPP and EHPHP [19]. All of the targeted bisphenols were detected in the samples. Three (BPF, BPA, and BPS) of the seven bisphenols were detected in > 50% of the samples. The most abundant bisphenol was BPA, followed by BPS and BPF [18].

Secular trends in the internal exposure to phthalates, PFRs, and bisphenols were investigated from 2012 to 2017. Despite updated phthalates regulations and reducing production volumes in Japan, all of the measured phthalate metabolites showed a stable trend between 2012 and 2017 [21]. Urinary levels of BDCIPP, BCIPHIPP, and EHPHP significantly increased by 13.3%, 12.9%, and 6.7% per year, respectively. Seasonal variations were observed with 2-fold higher levels of BCIPHIPP and BDCIPP in summer [19]. Furthermore, non-chlorinated PFR metabolites, EHPHP, BBOEP, and DPHP, were associated with increased levels of oxidative stress biomarkers, 8-OHdG. A PFR metabolite mixture was associated with increased levels of HEL, lipid hydroperoxide-modified lysine residue and HNE, a major fatty acid oxidation product, but not 8-OHdG [20].

The exposure level of BPA among 7-year-old children significantly decreased by 6.5% per year from 2012 to 2017, whereas BPS increased by 2.8% per year. The BPA and BPF levels were higher in children from low annual income households [18].

### Single nucleotide polymorphisms as genetic susceptibility

Here, we discuss new findings to understand the adverse health effects of gene–environment interaction for child outcomes (Table 6) based on an update in 2017 [9]. We first examined genetic vulnerability as a result of exposure to maternal smoking. We used the cotinine levels as the objective biomarkers to identify the maternal smoking status in the third trimester of pregnancy [76]. Using this data, we detected a dose-dependent association between the cotinine levels during pregnancy and reduced birth weight [25]. When considering the specific *aromatic hydrocarbon receptor* (*AHR*) (G > A; rs2066853) and *X-ray cross-complementing gene 1* (*XRCC1*) (C > T; rs1799782) genotypes in mothers, a greater birth weight reduction was observed among infants born to mothers with the highest cotinine levels [25]. Moreover, maternal passive smoking during pregnancy was associated with an increased risk of term-SGA [77]. The gene–environment interaction between maternal passive smoking during pregnancy and the *cytochrome P450* (*CYP*), *family 1, subfamily A, polypeptide 1* (*CYP1A1*; A>G; rs1051740) AG/GG genotype had an influence on the difference in the child head circumference up to 3 years of age [73].

**Table 6:** Environmental exposure during pregnancy and maternal and child outcomes: gene–environment interactions (only significant) in the literature after 2017 in the Hokkaido Study on Environment and Children’s Health

Environmental exposure during pregnancy	Maternal (M) or child (C) outcome	Maternal (M) or child (C) polymorphism	Maternal or child risk genotype	Change in the outcome	Ref.
Dioxin-like polychlorinated biphenyl (PCB)	(M: Dioxin-like PCB’s concentration)	M: *AHR* (G>A, Arg554Lys)	GA/AA	Concentration ↑	[67]
Dioxin and dioxin-like PCB	(M: Dioxin and dioxin-like PCB’s concentration)	M: *CYP1A1* (T>C; *MspI*)	TT/TC	Concentration ↑	[67]
Active smoking (based on questionnaire)	C: Reduction of birth weight	M: *AHR* (G>A, Arg554Lys)	Arg/Arg	Birth weight: 211 g ↓	[68]
Active smoking (based on questionnaire)	C: Reduction of birth weight	M: *CYP1A1* (m1/m2)	m1/m2 + m2/m2	170 g ↓	[68]
Active smoking (based on questionnaire)	C: Reduction of birth weight	M: *Combination of AHR* (G>A, Arg554Lys) and *CYP1A1* (m1/m2)	Combination of Arg/Arg (*AHR*) and m1/m2 + m2/m2 (*CYP1A1*)	315 g ↓	[68]
Active smoking (based on questionnaire)	C: Reduction of birth weight	M: Combination of *CYP1A1* (m1/m2) and *GSTM1* (non-null/null)	Combination of m1/m2 + m2/m2 (*CYP1A1*) and null (*GSTM1*)	237 g ↓	[68]
Active smoking (based on questionnaire)	C: Reduction of birth weight	M: *NQO1* (C>T, Pro187Ser)	Pro/Pro	159 g ↓	[69]
Active smoking (based on questionnaire)	C: Reduction of birth weight	M: *CYP2E1* (c1/c2)	c1/c1	195 g ↓	[69]
Active smoking (based on questionnaire)	C: Reduction of birth weight	M: *MTHFR* (A1298C)	AA	105.69 g ↓	[70]
Active smoking (based on cotinine level)	C: Reduction of birth weight	M: *CYP1A1* (A>G, Ile462Val)	AG/GG	62 g ↓	[71]
Active smoking (based on cotinine level)	C: Reduction of birth weight	M: *XRCC1* (C>T, Arg194Trp)	CT/TT	59 g ↓	[71]
Active smoking (based on cotinine level)	C: Reduction of birth weight	M: Combination of *AHR* (G>A, Arg554Lys), *CYP1A1* (A>G, Ile462Val), and *XRCC1* (C>T, Arg194Trp)	Combination of GG (*AHR*), AG/GG (*CYP1A1*), and CT/TT (*XRCC1*)	145 g ↓	[71]
Active smoking (based on cotinine level)	C: Reduction of birth weight	M: *AHR* (G>A, Arg554Lys)	GG	217 g ↓	[25]
Active smoking (based on cotinine level)	C: Reduction of birth weight	M: *XRCC1* (C>T, Arg194Trp)	TT	387 g ↓	[25]
Passive smoking (based on cotinine level)	C: Reduction of birth weight	M: *XRCC1* (C>T, Arg194Trp)	TT	139 g ↓	[25]
Dioxins	C: Reduction of birth weight	M: *GSTM1* (non-null/null)	Null	214 g ↓	[28]
Caffeine (≤300 mg/day)	C: Reduction of birth weight	M *CYP1A2* (C164A)	AA	277 g ↓	[72]
Passive smoking (based on cotinine level)	C: Reduction of head circumference gain from birth to 3 years	M: *CYP1A1* (A>G, Ile462Val)	AG/GG	0.75 cm ↓	[73]
The ratio of the lengths of the 2nd and 4th digits (2D/4D) which considered an index of prenatal androgen exposure at 7 years of age	(C: 2D/4D)	C: *ESR1* (A>G; rs9340799)	GG	2D/4D ↓	[74]
Mono(2-ethylhexyl) phthalate (MEHP) or Σ di(2-ethylhexyl) phthalate (DEHP)	C: 2D/4D	C: *ESR1* (A>G; rs2077647)	AG/GG	2D/4D ↓	[75]

↓, decreased; ↑, increased

Gene names: *AHR*, *aromatic hydrocarbon receptor*; *CYP1A1*, *cytochrome P450 1A1*; *CYP1A2*, *cytochrome P450 1A2*; *CYP2E1*, *cytochrome P450 2E1*; *ESR1*, *estrogen receptor 1*; *GSTM1*, *glutathione S-transferase mu 1*; *MTHFR*, *methylenetetrahydrofolate reductase*; *NQO1*, *NAD(P)H quinone oxidoreductase 1*; and *XRCC1*, *x-ray cross-complementing gene 1*

We also examined the effects of prenatal phthalate esters and BPA levels and the child *estrogen receptor 1* (*ESR1*) genotype on the ratio of the lengths of the second and fourth digits (2D/4D), which is considered to be an index of prenatal exposure to androgen among children at 7 years of age. Males with the *ESR1* (A > G; rs9340799) GG-genotype born to prenatal non-smokers had a significantly lower 2D/4D in the right hand than males with the AA/AG-genotype born to prenatal smokers [74]. Males with the *ESR1* (A > G; rs2077647) AG/GG genotype in the group exposed to high levels of MEHP or ΣDEHP showed feminized 2D/4D compared to males with the AA genotype who had low exposure to MEHP or ΣDEHP [75]. However, there were no associations between the mean 2D/4D and metabolites other than MEHP or BPA [75].

We observed that maternal and child genetic polymorphisms modify the effects that prenatal exposure to chemicals have on child outcomes from birth to childhood. Our investigations on gene–environment interactions suggest that there are high-risk genetic groups for the association between prenatal chemical exposure and child outcome. Hence, reducing exposure to specific chemicals in all pregnant women, including high-risk genetic groups, is important.

### Epigenetics as an underlying mechanism

One aspect of the underlying mechanisms in the early life environment and the resulting adverse health outcomes is epigenetic regulation. We first analyzed the DNA methylation levels among specific genes, insulin-like growth factor 2 (IGF2), H19, and long interspersed element 1 (LINE1), in cord blood. The associations between the PFAS and PCB levels in the maternal blood and DNA methylation levels were then examined. A notable positive association was found between IGF2 methylation and the PI values at birth. Moreover, 21% of a negative association between PFOA and the PI can be explained by the mediation of IGF2 gene methylation [28]. In contrast, among the PCBs, the DecaCB levels were positively associated with H19 gene DNA methylation and HeptaCBs were positively associated with global DNA methylation, LINE-1 [78]. We found an increase of 0.017 in the log10-transformed H19 methylation levels (%) in cord blood for each 10-fold increase in the DecaCB levels in maternal blood among all of the infants. Similarly, a 0.005 increase in the log10-transformed LINE-1 methylation levels (%) in cord blood was associated with a 10-fold increase in the HeptaCBs in maternal blood among all infants. In particular, we observed a dose-dependent association for the DecaCB levels in maternal blood with the H19 methylation levels among female infants; likewise, a dose-dependent association for the HeptaCBs levels was observed with the LINE-1 methylation levels among female infants. Moreover, these associations were only observed among infants born from primiparous women [78].

These results yielded new challenges. For example, using the genome-wide approach should provide a hypothesis-free assessment of the epigenetic alterations and environmental factors. Thus, as a next step, an epigenome-wide DNA methylation (EWAS) study was conducted using an Illumina Human Methylation 450 BeadChip (Illumina, Inc, San Diego, CA, USA) to quantify 485,577 CpGs’ DNA methylation. Prenatal exposure to PFOS, PFOA, and BPA, as well as their associations with differential methylation positions (DMPs) and regions (DMRs), was examined via robust linear regressions. As the Sapporo cohort is a discovery cohort, replicates were conducted by the Taiwan Maternal and Child Study (TMICS) [79] and the Taiwanese cohort [80]. When applying a false discovery rate of < 0.05 in the discovery cohort, four DMPs with the same direction in the replication cohort were found for PFOS while five DMPs were observed for PFOA. Methylation changes may plausibly occur in various signaling pathways, and this needs to be further studied to examine the persistence of DNA methylation changes due to prenatal PFAS exposure and the associations of these changes with health outcomes in longitudinal studies. For BPA, we observed that a significantly large portion of BPA-associated differentially methylated CpGs was hypomethylated in female infants (98%), as opposed to hypermethylation (88%) among male infants. Genes annotated to the FDR-corrected CpGs were clustered into an interconnected genetic network among males. In contrast, female-specific genes were significantly enriched in gene ontology (GO) terms related to cell adhesion. Our results suggest potential sex-specific epigenome responses to BPA exposure.

Associations between the DNA methylation and maternal smoking during pregnancy were also examined [81]. In this study, DNA methylation changes were compared among mothers who smoked until the second to third trimester, those who smoked but quit before the second to third trimester, and those who never smoked. Significant DNA hypermethylation was found among mothers who smoked until late into their pregnancy for ACSM3 (cg06478823), whereas hypomethylation among AHRR (cg05575921 and cg21161138) was found among mothers who smoked. The DNA methylation levels were similar for mothers who never smoked and those who quit smoking, suggesting that encouragement and support to stop smoking may be an effective technique to restore DNA methylation levels. To expand these findings, we selected the CpG sites of five genes, i.e., AHRR, CYP1A1, ESR1, MYO1G, and GF11, which showed significant DNA methylation alteration owing to maternal smoking during pregnancy. The DNA methylation levels were examined via next-generation sequencing t in a region of each gene. We examined whether the changes in DNA methylation mediate the association between prenatal smoking and ADHD symptoms, finding that DNA methylation of the *GF11* region mediated 48.4% of the total association between maternal active smoking during pregnancy and ADHD symptoms [82].

## Discussion

### Importance of longer follow-ups for prenatal exposure and later life examinations

**Fig. 2.**
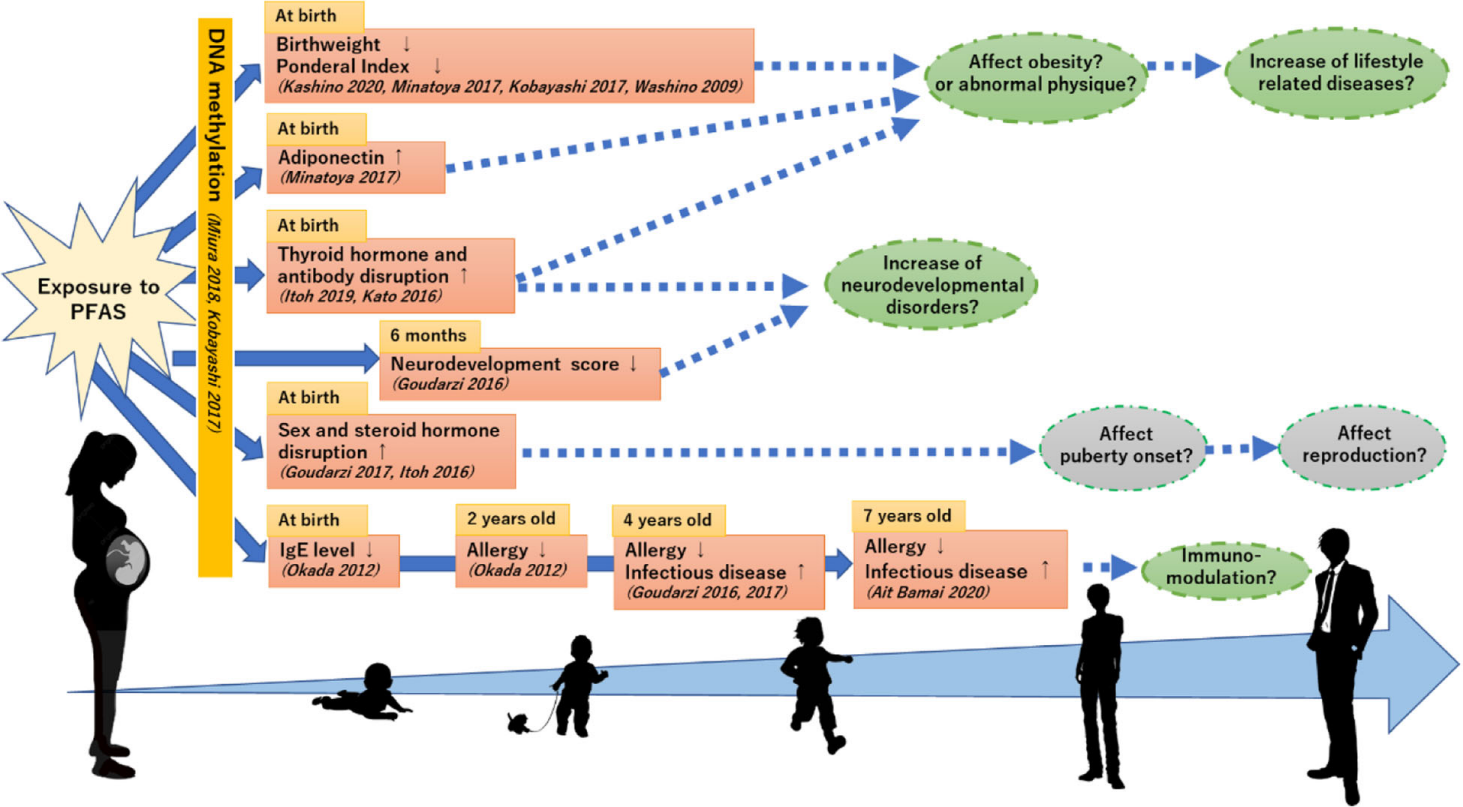
Importance of longer follow-ups to examine prenatal exposure and health impacts at later life. Solid arrows and squares indicate the health impacts of fetal and intrauterine exposure, respectively, to PFAS on the health of children based on the results observed across 16 reports published by the Hokkaido Study. For immuno-function, we observe continuous PFAS effects up to 7 years old. Lower IgE levels at birth; lower risk of allergies at 2, 4, and 7 years old; and higher risks of infectious disease at 4 and 7 years of age have also been observed. Ovals indicate the possible health impacts at later life stages based on the reported results

Recent studies have focused on the concept of the DOHaD hypothesis, which has spread internationally [83–85]. This hypothesis asserts that the state of a disease, such as obesity, diabetes, and cancer, later in adult life and throughout the life course is affected by environmental factors during prenatal and/or early postnatal periods [86]. Since the beginning of the Hokkaido Study, we have observed how maternal exposure to environmental chemicals, such as PCBs, dioxins, OCPs, and PFAS, affect children’s birth size, hormonal disruptions, and gene–environment interactions at birth [9]. In addition, maternal exposure affects postnatal neurodevelopment, infectious diseases, and allergies. We also observed associations between prenatal exposure to short half-life substances, such as phthalate and bisphenol, and neurodevelopment, hormonal disruption, and metabolic biomarkers [9, 39, 45]. We examined the effects that not only environmental exposure but also parental characteristics and SES during pregnancy have on birth weight, indicating the pathways between each factor via structural equation modeling [31, 32]. The Hokkaido Study reinforces the DOHaD hypothesis and proves the importance of preventive medicine. Figure 2 shows the possible impacts that fetal and intrauterine exposure to PFAS have on child health based on the results observed across the 16 reports published by the Hokkaido Study. As indicated by the squares in Fig. 2, the Hokkaido Study has previously revealed that maternal exposure to PFAS during pregnancy is significantly associated with children’s DNA methylation, physical growth, hormone status, neurobehavioral development, immune-functions, and reproductive functions from newborn to early childhood ages.

We should continue observation to examine how these findings shift and affect the future health status of these children through school, puberty, and adolescent ages, as shown in the Fig. 2. For example, our investigations of DNA methylation are based on the DOHaD approach. Maternal exposure to PFAS is associated with a reduced birth weight and PI, and increased adiponectin levels at birth. Low birth weight is a risk factor for an overweight or obesity status in later life stages, which is linked to lifestyle-related diseases, such as type 2 diabetes or cardiovascular disease, according to the DOHaD hypothesis. Exposure to PFAS during pregnancy is also associated with the disruption of the thyroid hormone and antibody levels at birth. The thyroid hormone, especially the T4 level, is related to physical growth and neurodevelopment during childhood. Further investigations should focus on how disrupted hormone levels at birth affect height, weight, and neurodevelopmental outcomes. In addition, we reported on the significant association between the maternal PFAS exposure level and neonatal reproductive hormone levels in cord blood. Whether reproductive hormones in utero or at birth are associated with the initiation of the puberty stage and reproductive functions in adulthood remains unclear. Continuous follow-ups should provide new data based on the DOHaD. For immune-function, the Hokkaido Study has previously reported results for lower IgE levels in cord blood, where reduced risks of allergies and increased risks of infectious diseases were found at follow-up ages of 2, 4, and 7 years. The duration of the long immune-modulative effect owing to PFAS exposure is the next issue to be examined in the Hokkaido Study. In addition, we must also focus not only prenatal exposure during critical periods, but also the influences of postnatal factors.

Previous studies have also suggested associations between maternal obesity and mental health problems associated with their children, where a possible mechanism is metabolic hormone-induced programming [87, 88]. In the Hokkaido cohort, we found a significant association between lower hyperactivity/inattention at pre-school age and higher cord leptin [58].

**Fig. 3:**
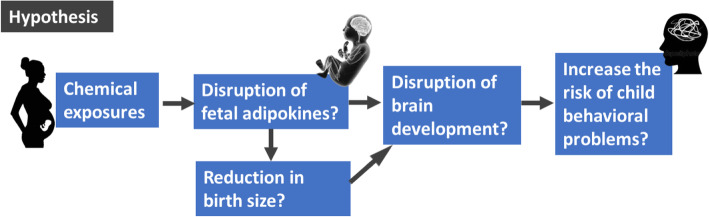
Hypothesis for the pathway of chemical exposure and child behavioral problems

Owing to the findings for prenatal exposure to environmental chemicals, which may decrease cord blood leptin levels that are associated with an increased risk of hyperactivity/inattention at pre-school age, we hypothesized that a decrease in cord leptin due to prenatal exposure to environmental chemicals may be linked to an increased risk of hyperactivity/inattention in children (Fig. 3). Further investigations are necessary to elucidate this hypothesis.

### Importance of examining secular trends in chemical exposure

**Fig. 4:**
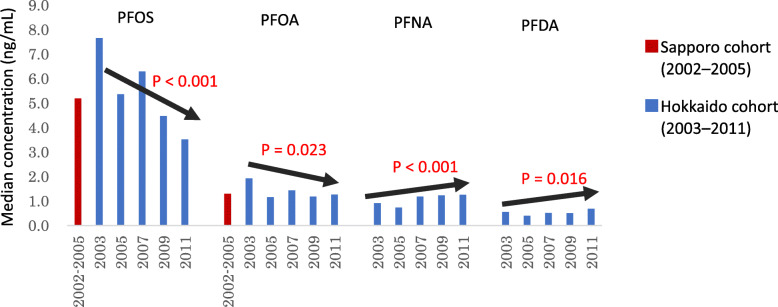
Secular trends of PFAS levels in maternal blood between the Sapporo and Hokkaido cohort

PFAS are POPs used in a wide range of consumer products [89]. The production of PFOS was restricted by Annex B of the 2009 Stockholm Convention on POPs. In addition, PFOA has been newly listed in Annex A of the Stockholm Convention on POPs. In the Sapporo cohort, we detected PFOS and PFOA concentrations of 5.2 and 1.3 ng/mL, respectively, in maternal blood, which were collected from 2002 to 2005. Maternal PFOS was associated with a decreasing birth weight [90]. PFAS with chains longer than those of PFOA have high bioconcentration factors, suggesting their environmental persistence [91]. Therefore, measuring the exposure levels of pregnant women to PFOS, PFOA, and other PFAS is necessary. Determining whether the environmental levels of these compounds change over time is also critical. In the Hokkaido cohort, 11 PFAS were measured; the concentrations of PFOS and PFOA in the plasma samples of pregnant women declined from 7.66 to 3.52 ng/mL and 1.93 to 1.27 ng/mL, respectively, from 2003 to 2011 (Fig. 4). These maternal PFOS and PFOA concentrations were not associated with birth weight. In contrast, the concentrations of perfluorononanoic acid (PFNA) and perfluorodecanoic acid (PFDA), which contain long-chain perfluorinated acids levels, increased from 2003 to 2011, as shown in Fig. 4 [15]. We observed negative associations among the 11 detected PFAS in maternal plasma at the third trimester and newborn birth size. A decrease in birth weight by 96.2 g and birth length by 0.48 cm per 10-fold increase in the PFNA concentration and a decrease in birth weight by 72.2 g per 10-fold increase in the PFDA concentration were noted [92].

### Importance of postnatal exposure assessment

We cannot ignore prenatal and postnatal exposures for children because humans are widely exposed to emerging contaminants present in products and foods, from baby care products and child toys to cosmetics, furniture, and textiles, as well as food contact materials throughout our lifetime. Furthermore, postnatal exposure to environmental chemicals from interior areas, diet, and consumer products may differ from those in the neonatal period as chemical production and uses change as a result of regulations and/or updated guidelines. Investigating the current internal exposure levels of shorter half-life compounds using urine samples has provided novel findings for Japanese children [18, 19, 21]. The internal exposure levels of certain phthalates and PFRs, which are used as plasticizers, flame retardants in polyurethane foam, and lubricants, are associated with indoor environments, such as various building types, floor materials, and ventilation habitats [19, 21, 93]. From 2012 to 2017, there was increase in the internal exposure levels of PFRs and BPS, alternatives for BPA, among children [18, 19], while there was a decrease in BPA levels [18]. Furthermore, the levels of phthalates did not show any secular trends during the study periods [21], suggesting that the exposure to shorter half-life environmental chemicals fluctuates according to governmental regulations and updated guidelines. Despite the fact that regulations and guidelines for limited consumer products and medical devices directly affect the internal exposure levels of such compounds, until now, there are no regulations or guidelines for building and/or interior materials. These materials and compounds, which are not currently considered as emergent environmental chemicals, therefore require continuous biomonitoring and risk assessments, including evaluations of their possible alternatives. Young children are an especially vulnerable population that are immature with respect to their development stage milestones. Moreover, humans are exposed to a mixture of chemicals originating from various sources daily. The combined effects of chemicals, as well as cumulative exposure to the same chemical from different sources, may enhance exposure reactions. Therefore, rigorous exposure assessments should be performed to account for chemical mixtures, cumulative exposure, and risk assessments.

Furthermore, the physiological environment may have multiple effects on child health. Several previous studies have investigated the association between exposure to the radio frequency electromagnetic field (RF-EMF), i.e., mobile phones, and child behavioral problems [94, 95] or cognitive functions [96–99]. To explore the association between such new types of exposure and child health, we conducted a survey on mobile phones or the internet environment and the health status of the children, including behavioral problems for all children in the cohort. In addition, we implemented three types of face-to-face surveys for evaluating neurodevelopment (cognitive assessment, electroencephalogram recording, and intelligence test), along with personal RF-EMF exposure measurements from portable devices, for a small proportion of the child participants (*n* = 400).

### Molecular biology in birth cohort

Numerous epidemiological studies on fetal exposure and outcomes have been published recently in Europe, North America, and Asia; however, few of these epidemiological studies consider fetal exposure and genetic factors [100, 101]. The Hokkaido birth cohort study is one of the leading studies to provide significant findings with such limited information. As listed in Table 6, the maternal SNP genotype underlies the associations among tobacco, caffeine, MEHP, and dioxin exposure for child outcomes after birth to 7 years of age, likely by modifying the elimination of toxic chemicals and their transfer to the fetus [25, 28, 68–70, 72, 73, 75]. Based on the results of the Hokkaido study, the genetic susceptibility of individuals to have varying birth sizes has become a clear modifier of fetal chemical exposure, which depends on the genotype of enzymatic-related genes in xenobiotic metabolism and that of related genes in disease development. Using our ongoing birth cohorts, we must examine the association of fetal chemical exposure and the maternal and child genotypes of them on child growth trajectory after birth in further studies. We speculate that the gene–environment interactions among the genotype of the gene that encodes the maternal chemical receptor, as well as the xenobiotic-metabolizing enzymes, and exposure to environmental chemicals during pregnancy affect the growth and neurodevelopment trajectories from birth to puberty. Therefore, examining the gene–environment interactions for the growth and neurodevelopment trajectories should be prioritized.

In addition, the results of the Hokkaido Study also suggest that certain chemicals alter DNA methylation in humans [79–82, 102]. Exposure to environmental chemicals during pregnancy causes child epigenome changes at specific sites in a wide range of functional genes at birth. Perhaps changes in the child epigenome may affect postnatal growth and neurodevelopment in the future. Therefore, we must examine whether the interaction between exposure to environmental chemicals and the child epigenome affects development after birth. Continuing the birth cohort study is essential to assess long-term health effects as a consequence of gene–environment interactions.

### Applying achievements to interventions

Through the Hokkaido Study birth cohort, we have demonstrated that the early life environment and chemical exposures affect child health; however, identifying determinants of exposure and environmental factors is not sufficient to change the life of a child. Based on the results obtained from epidemiological studies, elimination of harmful factors and provision of effective intervention are required to prevent later onset of diseases. The lifestyle, behavior, social class, and individual education are important factors that can determine and prevent health risks. We constructed a childhood obesity risk index based on predictors identified from pregnancy through the first year of life. The algorithm for the childhood obesity risk score at 6–8 years of age included the maternal pre-pregnancy BMI, gender of the child, smoking during pregnancy, education, and obesity index at 1 year old [103]. These predictive values can be used at pregnancy and newborn checkups to prevent child obesity, which is a risk factor for non-communicable diseases later in life.

## Conclusions

Twenty years have passed since the Hokkaido Study initiated the recruitment of pregnant women in 2002. In addition to a mail survey, we conducted face-to-face health checkups, home visits, and the collection of biospecimens from child participants. Different risk factors from parental characteristics on PTB, VLBW, and SGA were found. The maternal pre-pregnancy BMI, smoking, and alcohol consumption were found to independently mediate the effect on low SES to SGA. First-trimester maternal serum folate did not preclude a risk of birth defects. Prenatal chemical exposure and smoking were associated with birth size and growth, cord adiponectin and leptin, thyroid and reproductive hormones, neurodevelopment, and asthma and allergies. Chemical exposure can occur during the prenatal period and after birth. To reinforce the DOHaD hypothesis, longer follow-up periods are crucial. Additionally, considering all exposure levels is essential because the regulation of certain chemicals results in increased or decreased exposure levels, as exemplified by the secular trends found in this study. Exposure levels can thus change, in addition to the associations among health outcomes. This study found that individual susceptibility to adverse health effects depends on the genotype of enzymatic-related genes in xenobiotic metabolism. Epigenome modification of DNA methylation was also found to be consequence of prenatal chemical and tobacco exposure.

Global collaborations are ongoing, e.g., the consortium of Asian birth cohorts, the Birth Cohort Consortium of Asia [6], the Pregnancy And Childhood Epigenetics (PACE) [104], and the Consortium on Thyroid and Pregnancy [105]. Collaborations with multiple studies allow for joint-data analysis, meta-analysis, and increased statistical power to obtain robust findings. International collaborations add a new dimension to current knowledge and can lead to novel discoveries in the future.

## Data Availability

The datasets generated and/or analyzed during the current study are not publicly available because the study involves human participants with a nondisclosure provision of individual data stated in the written informed consent in order to prevent compromise of study participants’ privacy, but are available from the corresponding author upon reasonable request.

## Abbreviations

2D/4D: 2nd and 4th digits ratios
AA/GC: Adrenal androgens/glucocorticoid
ASD: Autism spectrum disorder
ADHD-RS: Attention Deficit Hyperactivity Disorder-Rating Scale
AHR: Aromatic hydrocarbon receptor
ART: Assisted reproductive technology
CEHS: Center for Environmental and Health Sciences
CYP: *Cytochrome P450*
CYP1A1: Cytochrome P450 1A1
DHEA: Dehydroepiandrosterone
DEHP: Di(2-ethylhexyl) phthalate
DMPs: Differentially methylation positions
DMRs: Differentially methylation regions
DOHaD: Developmental Origins of Health and Disease
E2: Estradiol
ESR1: *Estrogen receptor 1*
EWAS: Epigenome-wide association studies
FeNO: Fractional exhaled nitric oxide
FSH: Follicle stimulating hormone
FT4: Free thyroxine
GO: Gene ontology
IGF2: Insulin-like growth factor 2
ISAAC: International Study of Asthma and Allergies in Childhood
LINE1: Long interspersed element 1
MEHP: Mono(2-ethylhexyl) phthalate
MiBP: Mono-isobutyl phthalate
MnBP: Mono-n-butyl phthalate
OCPs: Organochlorine pesticides
OH-PCB: Hydroxylated polychlorinated biphenyl
P4: Progesterone
PCB: Polychlorinated biphenyl
PCDD: Polychlorinated dibenzo-p-dioxin
PCDF: Polychlorinated dibenzofuran
PFDA: Perfluorodecanoic acid
PFNA: Perfluorononanoic acid
PFOA: Perfluorooctanoic acid
PFOS: Perfluorooctanesulfonic acid
PFRs: Phosphate flame retardants
PI: Ponderal index
POPs: Persistent organic pollutants
PTB: Preterm-birth
RF-EMF: Radio frequency electromagnetic field
PRL: Prolactin
SCQ: Social Communication Questionnaire
SDQ: Strengths and Difficulties Questionnaire
SGA: Small for gestational age
SHBG: Steroid hormone binding globulin
SNP: Single nucleotide polymorphism
TA: Thyroid antibodies
TaqMan: Allelic discrimination assays, with fluorogenic probes and 5′ nuclease
T/E2: Testosterone/estradiol
term-SGA: Small for gestational age at full-term
TEQ: Toxicity equivalency quantity
TgAb: Thyroglobulin antibody
TH: Thyroid hormone
TMICS: Taiwan Maternal and Infant Cohort Study
TSH: Thyroid-stimulating hormone
VLBW: Very low birth weight
XRCC1: X-ray cross-complementing gene 1

## References

[1] WHO (2012). Endocrine disrupters and child health: Possible developmental early effects of endocrine disrupters on child health.

[2] Gluckman P, Hanson M. Early life origins of human health and disease. New York: Cambridge University Press; 2006. 10.1017/CBO9780511544699.

[3] Newman J, Ross M (2009). Early life origins of human health and disease.

[4] Kishi R, Grandgern F. (edited book) Health impacts of developmental exposure to environmental chemicals. Singapore: Springer; 2020.

[5] Vrijheid M, Casas M, Bergstrom A, Carmichael A, Cordier S, Eggesbo M (2012). European birth cohorts for environmental health research. Environ Health Perspect.

[6] Kishi R, Zhang J, Ha E, Chen P, Tian Y, Xia Y, et al. Birth Cohort Consortium of Asia (BiCCA) – current and future perspectives. Epidemiology. 2016;28(Suppl 1):S19–34. 10.1097/EDE.0000000000000698 29028672

[7] Kishi R, Sasaki S, Yoshioka E, Yuasa M, Sata F, Saijo Y, Kurahashi N, Tamaki J, Endo T, Sengoku K, Nonomura K, Minakami H, for the Hokkaido Study on Environment and Children's Health (2011). Cohort profile: the Hokkaido Study on Environment and Children’s Health in Japan. Int J Epidemiol.

[8] Kishi R, Kobayashi S, Ikeno T, Araki A, Miyashita C, Itoh S (2013). Ten years of progress in the Hokkaido birth cohort study on environment and children’s health: cohort profile—updated 2013. Environ Health Prev Med.

[9] Kishi R, Araki A, Minatoya M, Hanaoka T, Miyashita C, Itoh S (2017). The Hokkaido birth cohort study on environment and children’s health: Cohort profile – updated 2017. Environ Health Prev Med.

[10] Todaka T, Hirakawa H, Kajiwara J, Hori T, Tobiishi K, Onozuka D, Kato S, Sasaki S, Nakajima S, Saijo Y, Sata F, Kishi R, Iida T, Furue M (2007). Concentrations of polychlorinated dibenzo-p-dioxins, polychlorinated dibenzofurans, and dioxin-like polychlorinated biphenyls in blood collected from 195 pregnant women in Sapporo City, Japan. Chemosphere.

[11] Todaka T, Hirakawa H, Kajiwara J, Hori T, Tobiishi K, Onozuka D, Kato S, Sasaki S, Nakajima S, Saijo Y, Sata F, Kishi R, Iida T, Furue M (2008). Concentrations of polychlorinated dibenzo-p-dioxins, polychlorinated dibenzofurans, and dioxin-like polychlorinated biphenyls in blood and breast milk collected from 60 mothers in Sapporo City, Japan. Chemosphere.

[12] Todaka T, Hori T, Hirakawa H, Kajiwara J, Yasutake D, Onozuka D, Kato S, Sasaki S, Nakajima S, Saijo Y, Sata F, Kishi R, Iida T, Furue M (2008). Congener-specific analysis of non-dioxin-like polychlorinated biphenyls in blood collected from 195 pregnant women in Sapporo City, Japan. Chemosphere.

[13] Tobiishi K, Todaka T, Hirakawa H, Kajiwara J, Hirata T, Sasaki S (2009). Measurement method for hydroxylated polychlorinated biphenyls (OH-PCBs) in blood by LC/MS/MS. Organohalogen Compounds.

[14] Inoue K, Okada F, Ito R, Kato S, Sasaki S, Nakajima S, Uno A, Saijo Y, Sata F, Yoshimura Y, Kishi R, Nakazawa H (2004). Perfluorooctane sulfonate (PFOS) and related perfluorinated compounds in human maternal and cord blood samples: assessment of PFOS exposure in a susceptible population during pregnancy. Environ Health Perspect..

[15] Okada E, Kashino I, Matsuura H, Sasaki S, Miyashita C, Yamamoto J, Ikeno T, Ito YM, Matsumura T, Tamakoshi A, Kishi R (2013). Temporal trends of perfluoroalkyl acids in plasma samples of pregnant women in Hokkaido, Japan, 2003-2011. Environ Int.

[16] Kanazawa A, Miyasita C, Okada E, Kobayashi S, Washino N, Sasaki S, Yoshioka E, Mizutani F, Chisaki Y, Saijo Y, Kishi R (2012). Blood persistent organochlorine pesticides in pregnant women in relation to physical and environmental variables in The Hokkaido Study on Environment and Children's Health. Sci Total Environ.

[17] Araki A, Mitsui T, Miyashita C, Nakajima T, Naito H, Ito S, Sasaki S, Cho K, Ikeno T, Nonomura K, Kishi R (2014). Association between maternal exposure to di(2-ethylhexyl) phthalate and reproductive hormone levels in fetal blood: The Hokkaido Study on Environment and Children's Health. PLoS One..

[18] Gys C, Ait Bamai Y, Araki A, Bastiaensen M, Caballero-Casero N, Kishi R, Covaci A (2020). Biomonitoring and temporal trends of bisphenols exposure in Japanese school children. Environ Res.

[19] Bastiaensen M, Ait Bamai Y, Araki A, Goudarzi H, Konno S, Ito S, Miyashita C, Yao Y, Kishi R, Covaci A (2020). Temporal trends and determinants of PFR exposure in the Hokkaido Study. Int J Hyg Environ Health..

[20] Ait Bamai Y, Bastiaensen M, Araki A, Goudarzi H, Konno S, Ito S, Miyashita C, Yao Y, Covaci A, Kishi R (2019). Multiple exposures to organophosphate flame retardants alter urinary oxidative stress biomarkers among children: The Hokkaido Study. Environ Int.

[21] Ketema RM, Ait Bamai Y, Ikeda-Araki A, Saito T, Kishi R (2021). Secular trends of urinary phthalate metabolites in 7-year old children and association with building characteristics: Hokkaido study on environment and children's health. Int J Hyg Environ Health.

[22] Ait Bamai Y, Araki A, Nomura T, Kawai T, Tsuboi T, Kobayashi S, Miyashita C, Takeda M, Shimizu H, Kishi R (2018). Association of filaggrin gene mutations and childhood eczema and wheeze with phthalates and phosphorus flame retardants in house dust: The Hokkaido study on Environment and Children's Health. Environ Int..

[23] Ait Bamai Y, Araki A, Kawai T, Tsuboi T, Saito I, Yoshioka E (2014). Associations of phthalate concentrations in floor dust and multi-surface dust with the interior materials in Japanese dwellings. Sci Total Environ.

[24] Tajima S, Araki A, Kawai T, Tsuboi T, Ait Bamai Y, Yoshioka E, Kanazawa A, Cong S, Kishi R (2014). Detection and intake assessment of organophosphate flame retardants in house dust in Japanese dwellings. Sci Total Environ.

[25] Kobayashi S, Sata F, Sasaki S, Braimoh TS, Araki A, Miyashita C, Goudarzi H, Kobayashi S, Kishi R (2017). Modification of adverse health effects of maternal active and passive smoking by genetic susceptibility: Dose-dependent association of plasma cotinine with infant birth size among Japanese women-The Hokkaido Study. Reprod Toxicol.

[26] Spurgeon SL, Jones RC, Ramakrishnan R (2008). High throughput gene expression measurement with real time PCR in a microfluidic dynamic array. PLoS One.

[27] Wang J, Lin M, Crenshaw A, Hutchinson A, Hicks B, Yeager M, Berndt S, Huang WY, Hayes RB, Chanock SJ, Jones RC, Ramakrishnan R (2009). High-throughput single nucleotide polymorphism genotyping using nanofluidic Dynamic Arrays. BMC Genomics.

[28] Kobayashi S, Azumi K, Goudarzi H, Araki A, Miyashita C, Kobayashi S, Itoh S, Sasaki S, Ishizuka M, Nakazawa H, Ikeno T, Kishi R (2016). Effects of prenatal perfluoroalkyl acid exposure on cord blood IGF2/H19 methylation and ponderal index: The Hokkaido Study. J Expo Sci Environ Epidemiol.

[29] Bibikova M, Barnes B, Tsan C, Ho V, Klotzle B, Le JM (2011). High density DNA methylation array with single CpG site resolution. Genomics.

[30] Sandoval J, Heyn H, Moran S, Serra-Musach J, Pujana MA, Bibikova M, Esteller M (2011). Validation of a DNA methylation microarray for 450,000 CpG sites in the human genome. Epigenetics.

[31] Tamura N, Hanaoka T, Ito K, Araki A, Miyashita C, Ito S, Minakami H, Cho K, Endo T, Sengoku K, Ogasawara K, Kishi R (2018). Different risk factors for very low birth weight, term-small-for-gestational-age, or preterm birth in Japan. Int J Environ Res Public Health.

[32] Tamura N, Hanaoka T, Ito K, Araki A, Miyashita C, Ito S, Kobayashi S, Ito Y, Minakami H, Cho K, Endo T, Baba T, Sengoku K, Miyamoto T, Ogasawara K, Kishi R (2021). Mediating factors between parental socioeconomic status and small for gestational age in infants: results from the Hokkaido Study on Environment and Children's Health. Matern Child Health J.

[33] Poudel K, Kobayashi S, Miyashita C, Ikeda-Araki A, Tamura N, Ait Bamai Y, Itoh S, Yamazaki K, Masuda H, Itoh M, Ito K, Kishi R (2021). Hypertensive disorders during pregnancy (HDP), maternal characteristics, and birth outcomes among Japanese women: a Hokkaido study. Int J Environ Res Public Health..

[34] Hanaoka T, Tamura N, Ito K, Sasaki S, Araki A, Ikeno T, Miyashita C, Ito S, Minakami H, Cho K, Endo T, Baba T, Miyamoto T, Sengoku K, Kishi R, other members of the Hokkaido Study on Environment and Children’s Health (2018). Prevalence and risk of birth defects observed in a prospective cohort study: The Hokkaido Study on Environment and Children's Health. J Epidemiol.

[35] Ito K, Hanaoka T, Tamura N, Sasaki S, Miyashita C, Araki A, Ito S, Minakami H, Cho K, Endo T, Baba T, Miyamoto T, Sengoku K, Tamakoshi A, Kishi R (2019). Association between maternal serum folate concentrations in the first trimester and the risk of birth defects: The Hokkaido Study of Environment and Children's Health. J Epidemiol.

[36] Srikanthan K, Feyh A, Visweshwar H, Shapiro JI, Sodhi K (2016). Systematic review of metabolic syndrome biomarkers: a panel for early detection, management, and risk stratification in the West Virginian population. Int J Med Sci.

[37] Minatoya M, Itoh S, Miyashita C, Araki A, Sasaki S, Miura R, Goudarzi H, Iwasaki Y, Kishi R (2017). Association of prenatal exposure to perfluoroalkyl substances with cord blood adipokines and birth size: The Hokkaido Study on environment and children's health. Environ Res.

[38] Minatoya M, Araki A, Miyashita C, Sasaki S, Goto Y, Nakajima T, Kishi R (2017). Prenatal di-2-ethylhexyl phthalate exposure and cord blood adipokine levels and birth size: The Hokkaido study on environment and children's health. Sci Total Environ.

[39] Minatoya M, Araki A, Miyashita C, Ait Bamai Y, Itoh S, Yamamoto J, Onoda Y, Ogasawara K, Matsumura T, Kishi R (2018). Association between prenatal bisphenol A and phthalate exposures and fetal metabolic related biomarkers: The Hokkaido study on Environment and Children's Health. Environ Res..

[40] Baba T, Ito S, Yuasa M, Yoshioka E, Miyashita C, Araki A, Sasaki S, Kobayashi S, Kajiwara J, Hori T, Kato S, Kishi R (2018). Association of prenatal exposure to PCDD/Fs and PCBs with maternal and infant thyroid hormones: The Hokkaido Study on Environment and Children's Health. Sci Total Environ.

[41] Itoh S, Baba T, Yuasa M, Miyashita C, Kobayashi S, Araki A, Sasaki S, Kajiwara J, Hori T, Todaka T, Fujikura K, Nakajima S, Kato S, Kishi R (2018). Association of maternal serum concentration of hydroxylated polychlorinated biphenyls with maternal and neonatal thyroid hormones: The Hokkaido birth cohort study. Environ Res.

[42] Yamazaki K, Itoh S, Araki A, Miyashita C, Minatoya M, Ikeno T, Kato S, Fujikura K, Mizutani F, Chisaki Y, Kishi R (2020). Associations between prenatal exposure to organochlorine pesticides and thyroid hormone levels in mothers and infants: The Hokkaido study on environment and children's health. Environ Res.

[43] Kato S, Itoh S, Yuasa M, Baba T, Miyashita C, Sasaki S, Nakajima S, Uno A, Nakazawa H, Iwasaki Y, Okada E, Kishi R (2016). Association of perfluorinated chemical exposure in utero with maternal and infant thyroid hormone levels in the Sapporo cohort of Hokkaido Study on the Environment and Children's Health. Environ Health Prev Med.

[44] Minatoya M, Nakajima S, Sasaki S, Araki A, Miyashita C, Ikeno T (2016). Effects of prenatal phthalate exposure on thyroid hormone levels, mental and psychomotor development of infants: The Hokkaido Study on Environment and Children's Health. Sci Total Environ.

[45] Minatoya M, Sasaki S, Araki A, Miyashita C, Itoh S, Yamamoto J, Matsumura T, Mitsui T, Moriya K, Cho K, Morioka K, Minakami H, Shinohara N, Kishi R (2017). Cord Blood bisphenol A levels and reproductive and thyroid hormone levels of neonates: The Hokkaido Study on Environment and Children's Health. Epidemiology.

[46] Itoh S, Araki A, Miyashita C, Yamazaki K, Goudarzi H, Minatoya M (2019). Association between perfluoroalkyl substance exposure and thyroid hormone/thyroid antibody levels in maternal and cord blood: The Hokkaido Study. Environ Int.

[47] Miyashita C, Araki A, Mitsui T, Itoh S, Goudarzi H, Sasaki S, Kajiwara J, Hori T, Cho K, Moriya K, Shinohara N, Nonomura K, Kishi R (2018). Sex-related differences in the associations between maternal dioxin-like compounds and reproductive and steroid hormones in cord blood: The Hokkaido study. Environ Int.

[48] Araki A, Miyashita C, Mitsui T, Goudarzi H, Mizutani F, Chisaki Y, Itoh S, Sasaki S, Cho K, Moriya K, Shinohara N, Nonomura K, Kishi R (2018). Prenatal organochlorine pesticide exposure and the disruption of steroids and reproductive hormones in cord blood: The Hokkaido study. Environ Int.

[49] Itoh S, Araki A, Mitsui T, Miyashita C, Goudarzi H, Sasaki S, Cho K, Nakazawa H, Iwasaki Y, Shinohara N, Nonomura K, Kishi R (2016). Association of perfluoroalkyl substances exposure in utero with reproductive hormone levels in cord blood in the Hokkaido Study on Environment and Children's Health. Environ Int.

[50] Goudarzi H, Araki A, Itoh S, Sasaki S, Miyashita C, Mitsui T, Nakazawa H, Nonomura K, Kishi R (2017). The association of prenatal exposure to perfluorinated chemicals with glucocorticoid and androgenic hormones in cord blood samples: the Hokkaido study. Environ Health Perspect.

[51] Araki A, Mitsui T, Goudarzi H, Nakajima T, Miyashita C, Itoh S, Sasaki S, Cho K, Moriya K, Shinohara N, Nonomura K, Kishi R (2017). Prenatal di(2-ethylhexyl) phthalate exposure and disruption of adrenal androgens and glucocorticoids levels in cord blood: The Hokkaido Study. Sci Total Environ.

[52] Mitsui T, Araki A, Goudarzi H, Miyashita C, Ito S, Sasaki S, Kitta T, Moriya K, Cho K, Morioka K, Kishi R, Shinohara N, Takeda M, Nonomura K (2018). Relationship between adrenal steroid hormones in cord blood and birth weight: The Sapporo Cohort, Hokkaido Study on Environment and Children's Health. Am J Hum Biol.

[53] Yamazaki K, Araki A, Nakajima S, Miyashita C, Ikeno T, Itoh S, Minatoya M, Kobayashi S, Mizutani F, Chisaki Y, Kishi R (2018). Association between prenatal exposure to organochlorine pesticides and the mental and psychomotor development of infants at ages 6 and 18 months: The Hokkaido Study on Environment and Children's Health. Neurotoxicology.

[54] Ikeno T, Miyashita C, Nakajima S, Kobayashi S, Yamazaki K, Saijo Y, Kita T, Sasaki S, Konishi K, Kajiwara J, Hori T, Kishi R (2018). Effects of low-level prenatal exposure to dioxins on cognitive development in Japanese children at 42months. Sci Total Environ.

[55] Minatoya M, Itoh S, Yamazaki K, Araki A, Miyashita C, Tamura N, Yamamoto J, Onoda Y, Ogasawara K, Matsumura T, Kishi R (2018). Prenatal exposure to bisphenol A and phthalates and behavioral problems in children at preschool age: the Hokkaido Study on Environment and Children's Health. Environ Health Prev Med.

[56] Minatoya M, Araki A, Itoh S, Yamazaki K, Kobayashi S, Miyashita C, Sasaki S, Kishi R (2019). Prenatal tobacco exposure and ADHD symptoms at pre-school age: the Hokkaido Study on Environment and Children's Health. Environ Health Prev Med.

[57] Suyama S, Yagyu K, Araki A, Miyashita C, Itoh S, Minatoya M, Yamazaki K, Tamura N, Nakai A, Saito T, Kishi R (2020). Risk factors for motor coordination problems in preschool-age children. Pediatr Int.

[58] Minatoya M, Itoh S, Araki A, Tamura N, Yamazaki K, Miyashita C, et al. Association between fetal adipokines and child behavioral problems at preschool age: The Hokkaido Study on Environment and Children's Health. Int J Environ Res Public Health. 2018;15(1):120. https://www.ncbi.nlm.nih.gov/pmc/articles/PMC5800219/pdf/ijerph-15-00120.pdf. 10.3390/ijerph15010120 PMC5800219 29324697

[59] Okada E, Sasaki S, Kashino I, Matsuura H, Miyashita C, Kobayashi S, Itoh K, Ikeno T, Tamakoshi A, Kishi R (2014). Prenatal exposure to perfluoroalkyl acids and allergic diseases in early childhood. Environ Int.

[60] Goudarzi H, Miyashita C, Okada E, Kashino I, Kobayashi S, Chen CJ, Ito S, Araki A, Matsuura H, Ito YM, Kishi R (2016). Effects of prenatal exposure to perfluoroalkyl acids on prevalence of allergic diseases among 4-year-old children. Environ Int.

[61] Ait Bamai Y, Goudarzi H, Araki A, Okada E, Kashino I, Miyashita C, Kishi R (2020). Effect of prenatal exposure to per- and polyfluoroalkyl substances on childhood allergies and common infectious diseases in children up to age 7 years: The Hokkaido study on environment and children's health. Environ Int.

[62] Goudarzi H, Miyashita C, Okada E, Kashino I, Chen C-J, Ito S, Araki A, Kobayashi S, Matsuura H, Kishi R (2017). Prenatal exposure to perfluoroalkyl acids and prevalence of infectious diseases up to 4 years of age. Environ Int.

[63] Okada E, Sasaki S, Saijo Y, Washino N, Miyashita C, Kobayashi S, Konishi K, Ito YM, Ito R, Nakata A, Iwasaki Y, Saito K, Nakazawa H, Kishi R Prenatal exposure to perfluorinated chemicals and relationship with allergies and infectious diseases in infants. Environ Res 2012;112(0):118-125, DOI: 10.1016/j.envres.2011.10.003. 10.1016/j.envres.2011.10.003 22030285

[64] Miyashita C, Ait Bamai Y, Araki A, Itoh S, Minatoya M, Kobayashi S (2018). Prenatal exposure to dioxin-like compounds is associated with decreased cord blood IgE and increased risk of wheezing in children aged up to 7years: The Hokkaido study. Sci Total Environ.

[65] Ait Bamai Y, Miyashita C, Araki A, Nakajima T, Sasaki S, Kishi R (2018). Effects of prenatal di(2-ethylhexyl) phthalate exposure on childhood allergies and infectious diseases: The Hokkaido Study on Environment and Children's Health. Sci Total Environ.

[66] Goudarzi H, Konno S, Kimura H, Araki A, Miyashita C, Itoh S, Ait Bamai Y, Kimura H, Shimizu K, Suzuki M, Ito YM, Nishimura M, Kishi R (2018). Contrasting associations of maternal smoking and pre-pregnancy BMI with wheeze and eczema in children. Sci Total Environ.

[67] Kobayashi S, Sata F, Sasaki S, Ban S, Miyashita C, Okada E, Limpar M, Yoshioka E, Kajiwara J, Todaka T, Saijo Y, Kishi R (2013). Genetic association of aromatic hydrocarbon receptor (AHR) and cytochrome P450, family 1, subfamily A, polypeptide 1 (CYP1A1) polymorphisms with dioxin blood concentrations among pregnant Japanese women. Toxicol Lett.

[68] Sasaki S, Kondo T, Sata F, Saijo Y, Katoh S, Nakajima S, Ishizuka M, Fujita S, Kishi R (2006). Maternal smoking during pregnancy and genetic polymorphisms in the Ah receptor, CYP1A1 and GSTM1 affect infant birth size in Japanese subjects. Mol Hum Reprod.

[69] Sasaki S, Sata F, Katoh S, Saijo Y, Nakajima S, Washino N, Konishi K, Ban S, Ishizuka M, Kishi R (2008). Adverse birth outcomes associated with maternal smoking and polymorphisms in the N-Nitrosamine-metabolizing enzyme genes NQO1 and CYP2E1. Am J Epidemiol.

[70] Yila TA, Sasaki S, Miyashita C, Braimoh TS, Kashino I, Kobayashi S, Okada E, Baba T, Yoshioka E, Minakami H, Endo T, Sengoku K, Kishi R (2012). Effects of maternal 5,10-methylenetetrahydrofolate reductase C677T and A1298C Polymorphisms and tobacco smoking on infant birth weight in a Japanese population. J Epidemiol.

[71] Kobayashi S, Sata F, Miyashita C, Sasaki S, Ban S, Araki A (2016). Dioxin-metabolizing genes in relation to effects of prenatal dioxin levels and reduced birth size: the Hokkaido study. Reprod Toxicol.

[72] Sasaki S, Limpar M, Sata F, Kobayashi S, Kishi R (2017). Interaction between maternal caffeine intake during pregnancy and CYP1A2 C164A polymorphism affects infant birth size in the Hokkaido study. Pediatr Res.

[73] Braimoh TS, Kobayashi S, Sata F, Sasaki S, Goudarzi H, Yila TA (2017). Association of prenatal passive smoking and metabolic gene polymorphisms with child growth from birth to 3years of age in the Hokkaido Birth Cohort Study on Environment and Children's Health. Sci Total Environ.

[74] Nishimura Y, Moriya K, Kobayashi S, Araki A, Sata F, Mitsui T, Itoh S, Miyashita C, Cho K, Kon M, Nakamura M, Kitta T, Murai S, Kishi R, Shinohara N (2019). Association between ESR1 polymorphisms and second to fourth digit ratio in school-aged children in the Hokkaido Study. Steroids.

[75] Nishimura Y, Moriya K, Kobayashi S, Araki A, Sata F, Mitsui T, Itoh S, Miyashita C, Cho K, Kon M, Nakamura M, Kitta T, Murai S, Kishi R, Shinohara N (2020). Association of exposure to prenatal phthalate esters and bisphenol A and polymorphisms in the ESR1 gene with the second to fourth digit ratio in school-aged children: Data from the Hokkaido study. Steroids.

[76] Sasaki S, Braimoh TS, Yila TA, Yoshioka E, Kishi R (2011). Self-reported tobacco smoke exposure and plasma cotinine levels during pregnancy - a validation study in Northern Japan. Sci Total Environ.

[77] Kobayashi S, Sata F, Hanaoka T, Braimoh TS, Ito K, Tamura N, Araki A, Itoh S, Miyashita C, Kishi R (2019). Association between maternal passive smoking and increased risk of delivering small-for-gestational-age infants at full-term using plasma cotinine levels from The Hokkaido Study: a prospective birth cohort. BMJ Open.

[78] Kobayashi S, Sata F, Miyashita C, Miura R, Azumi K, Kobayashi S, et al. Gender-specific association of exposure to non-dioxin-like polychlorinated biphenyls during pregnancy with methylation levels of H19 and long interspersed nuclear element-1 in cord blood in the Hokkaido study. Toxicology. 2017;390:135–45. 10.1016/j.tox.2017.08.010 28865728

[79] Miura R, Araki A, Miyashita C, Kobayashi S, Kobayashi S, Wang SL, Chen CH, Miyake K, Ishizuka M, Iwasaki Y, Ito YM, Kubota T, Kishi R (2018). An epigenome-wide study of cord blood DNA methylations in relation to prenatal perfluoroalkyl substance exposure: The Hokkaido study. Environ Int.

[80] Miura R, Araki A, Minatoya M, Miyake K, Chen ML, Kobayashi S, Miyashita C, Yamamoto J, Matsumura T, Ishizuka M, Kubota T, Kishi R (2019). An epigenome-wide analysis of cord blood DNA methylation reveals sex-specific effect of exposure to bisphenol A. Sci Rep.

[81] Miyake K, Kawaguchi A, Miura R, Kobayashi S, Tran NQV, Kobayashi S, Miyashita C, Araki A, Kubota T, Yamagata Z, Kishi R (2018). Association between DNA methylation in cord blood and maternal smoking: The Hokkaido Study on Environment and Children's Health. Sci Rep.

[82] Miyake K, Miyashita C, Ikeda-Araki A, Miura R, Itoh S, Yamazaki K, Kobayashi S, Masuda H, Ooka T, Yamagata Z, Kishi R (2021). DNA methylation of GFI1 as a mediator of the association between prenatal smoking exposure and ADHD symptoms at 6 years: the Hokkaido Study on Environment and Children's Health. Clin Epigenetics..

[83] Mandy M, Nyirenda M (2018). Developmental Origins of Health and Disease: the relevance to developing nations. International Health..

[84] Suzuki K (2018). The developing world of DOHaD. J Dev Orig Health Dis..

[85] Gage SH, Munafò MR, Davey SG (2016). Causal Inference in Developmental Origins of Health and Disease (DOHaD) Research. Annu Rev Psychol.

[86] Barker DJ (2007). The origins of the developmental origins theory. J Int Med.

[87] Camargos AC, Mendonça VA, Oliveira KS, de Andrade CA, Leite HR, da Fonseca SF (2017). Association between obesity-related biomarkers and cognitive and motor development in infants. Behav Brain Res.

[88] Ashley-Martin J, Dodds L, Arbuckle TE, Ettinger AS, Shapiro GD, Fisher M, Morisset AS, Taback S, Bouchard MF, Monnier P, Dallaire R, Fraser WD (2014). A birth cohort study to investigate the association between prenatal phthalate and bisphenol A exposures and fetal markers of metabolic dysfunction. Environ Health Glob Access Sci Source.

[89] Lau C, Anitole K, Hodes C, Lai D, Pfahles-Hutchens A, Seed J (2007). Perfluoroalkyl acids: a review of monitoring and toxicological findings. Toxicol Sci.

[90] Washino N, Saijo Y, Sasaki S, Kato S, Ban S, Konishi K, Ito R, Nakata A, Iwasaki Y, Saito K, Nakazawa H, Kishi R (2009). Correlations between prenatal exposure to perfluorinated chemicals and reduced fetal growth. Environ Health Perspect.

[91] Martin JW, Mabury SA, Solomon KR, Muir DC (2003). Bioconcentration and tissue distribution of perfluorinated acids in rainbow trout (Oncorhynchus mykiss). Environ Toxicol Chem.

[92] Kashino I, Sasaki S, Okada E, Matsuura H, Goudarzi H, Miyashita C, Okada E, Ito YM, Araki A, Kishi R (2020). Prenatal exposure to 11 perfluoroalkyl substances and fetal growth: A large-scale, prospective birth cohort study. Environ Int.

[93] Bastiaensen M, Ait Bamai Y, Araki A, Van den Eede N, Kawai T, Tsuboi T (2019). Biomonitoring of organophosphate flame retardants and plasticizers in children: Associations with house dust and housing characteristics in Japan. Environ Res.

[94] Roser K, Schoeni A, Röösli M (2016). Mobile phone use, behavioural problems and concentration capacity in adolescents: A prospective study. Int J Hyg Environ Health.

[95] Byun YH, Ha M, Kwon HJ, Hong YC, Leem JH, Sakong J, Kim SY, Lee CG, Kang D, Choi HD, Kim N (2013). Mobile phone use, blood lead levels, and attention deficit hyperactivity symptoms in children: a longitudinal study. PLoS One..

[96] Brzozek C, Benke KK, Zeleke BM, Croft RJ, Dalecki A, Dimitriadis C, Kaufman J, Sim MR, Abramson MJ, Benke G (2019). Uncertainty Analysis of Mobile Phone Use and Its Effect on Cognitive Function: The Application of Monte Carlo Simulation in a Cohort of Australian Primary School Children. Int J Environ Res Public Health.

[97] Foerster M, Thielens A, Joseph W, Eeftens M, Röösli M (2018). A prospective cohort study of adolescents’ memory performance and individual brain dose of microwave radiation from wireless communication. Environ Health Perspect.

[98] Schoeni A, Roser K, Röösli M (2015). Memory performance, wireless communication and exposure to radiofrequency electromagnetic fields: a prospective cohort study in adolescents. Environ Int.

[99] Thomas S, Benke G, Dimitriadis C, Inyang I, Sim MR, Wolfe R, Croft RJ, Abramson MJ (2010). Use of mobile phones and changes in cognitive function in adolescents. Occup Environ Med.

[100] Rumrich I, Vähäkangas K, Viluksela M, Gissler M, de Ruyter H, Hänninen O (2020). Effects of maternal smoking on body size and proportions at birth: a register-based cohort study of 1.4 million births. BMJ Open.

[101] Yan J, Groothuis PA (2015). Timing of prenatal smoking cessation or reduction and infant birth weight: evidence from the United Kingdom Millennium Cohort Study. Matern Child Health J.

[102] Miura R, Ikeda-Araki A, Ishihara T, Miyake K, Miyashita C, Nakajima T, Kobayashi S, Ishizuka M, Kubota T, Kishi R (2021). Effect of prenatal exposure to phthalates on epigenome-wide DNA methylations in cord blood and implications for fetal growth: The Hokkaido Study on Environment and Children's Health. Sci Total Environ.

[103] Saijo Y, Ito Y, Yoshioka E, Sato Y, Minatoya M, Araki A, Miyashita C, Kishi R (2019). Identifying a risk score for childhood obesity based on predictors identified in pregnant women and 1-year-old infants: An analysis of the data of the Hokkaido Study on Environment and Children's Health. Clin Pediatr Endocrinol.

[104] Felix JF, Joubert BR, Baccarelli AA, Sharp GC, Almqvist C, Annesi-Maesano I (2018). Cohort Profile: Pregnancy And Childhood Epigenetics (PACE) Consortium. Int J Epidemiol.

[105] Korevaar TIM, Dhillon-Smith R, Coomarasamy A, Peeters RP (2019). An Invitation to Collaborate in the Consortium on Thyroid and Pregnancy. Eur Thyroid J.

